# Ethnic discrimination unlearned: experience in the repeated Trust Game reduces trust bias

**DOI:** 10.3389/fpsyg.2023.1139128

**Published:** 2023-05-25

**Authors:** Caitlin Duncan, Ulf Tölch, Henrik Walter, Isabel Dziobek

**Affiliations:** ^1^School of Mind and Brain, Humboldt-Universität zu Berlin, Berlin, Germany; ^2^Institute for Psychology, Humboldt-Universität zu Berlin, Berlin, Germany; ^3^Berlin Institute of Health (BIH) at Charité, BIH Quest Center for Responsible Research, Berlin, Germany; ^4^Department of Psychiatry and Neurosciences, Charité – Universitätsmedizin Berlin, Berlin, Germany

**Keywords:** Trust Game, ethnic bias, trust learning, reinforcement learning models, ingroup outgroup

## Abstract

**Introduction:**

Discrimination toward ethnic minorities is a persistent societal problem. One reason behind this is a bias in trust: people tend to trust their ingroup and comparatively distrust outgroups.

**Methods:**

In this study, we investigated whether and how people change their explicit trust bias with respect to ethnicity based on behavioral interactions with in- and outgroup members in a modified Trust Game.

**Results:**

Subjects’ initial explicit trust bias disappeared after the game. The change was largest for ingroup members who behaved unfairly, and the reduction of trust bias generalized to a small sample of new in- and outgroup members. Reinforcement learning models showed subjects’ learning was best explained by a model with only one learning rate, indicating that subjects learned from trial outcomes and partner types equally during investment.

**Discussion:**

We conclude that subjects can reduce bias through simple learning, in particular by learning that ingroup members can behave unfairly.

## Introduction

Trust can be defined as a person’s willingness to be vulnerable with another person while expecting a positive outcome from sharing that vulnerability (Colquitt et al., 2007). As such, trust involves some level of risk, but also an expectation or belief that a positive outcome will result. Trust can be split into two types: (1) attitudinal or general trust and (2) interpersonal or person-specific trust (Yamagishi, 2011; Yamagishi et al., 2015). Specifically, Yamagishi (2011) defines general trust as a default trust in other people when sufficient information is missing to judge whether they are trustworthy or not. Interpersonal, or person-specific trust, on the other hand, develops over time through experience and interaction with another individual (Yamagishi, 2011). Importantly, general trust is replaced by interpersonal trust – the expectations of trustworthiness of a particular individual - through repeated interaction with that individual (Yamagishi, 2011; Yamagishi et al., 2015).

Importantly, when minimal knowledge about an individual is available, trust is subject to biases; that is, the same level of trust is not applied to all unknown individuals equally. In the context of group membership, people extend more trust to ingroup members than to outgroup members and perceive them to be more trustworthy than outgroup members (meta-analysis: Balliet et al., 2014). Evidence from cooperation games shows that participants demonstrate an ingroup preference both trusting and cooperating more with ingroup members than with outgroup members, and additionally, are more likely to incur a personal cost to benefit ingroup members than to do the same for outgroup members (Balliet et al., 2014; review: Everett et al., 2015; Romano et al., 2017).^1^ This ingroup trust bias has been shown to be driven by ingroup-favoritism as opposed to outgroup-derogation (Balliet et al., 2014), reflecting the role of ingroup-favoritism in discrimination more broadly (review: Greenwald and Pettigrew, 2014).

When considering interpersonal trust learning, findings from trust learning literature have demonstrated that individuals’ trust biases (e.g., perceiving one partner to be much more trustworthy than another) can be changed through a multi-round Trust Game in which participants learn about their partners’ true trustworthiness (Chang et al., 2010; Fareri et al., 2012, 2015; Vermue et al., 2018). The Trust Game is an extensively researched paradigm for measuring trust as a behavior rather than self-report. In the classic Trust Game (Camerer and Weigelt, 1988; Berg et al., 1995), there are two players: a trustor and a trustee. The trustor is given a monetary endowment and asked to decide how much to transfer to the trustee. The amount they transfer is multiplied by the experimenter. The trustee is then asked to decide how much of the multiplied amount to send back to the trustor. In a multi-round version, participants play the game with the same partner(s) multiple times, so they can learn their reputation(s) (Phan et al., 2010). As such, the repeated Trust Game is an ideal paradigm to measure how trust with minimal knowledge about others, is then replaced by individual trust. Prior repeated TG research has shown that participants update their perceptions of their partners’ trustworthiness through repeated interactions in the TG, although in some cases it does not change participants’ trust bias entirely (Chang et al., 2010; Fareri et al., 2012, 2015; Fujino et al., 2020).

With respect to trust bias in intergroup contexts, Vermue et al. (2018) found across 3 experiments that participants rated nationality-based outgroup members as more trustworthy than ingroup members at the start of the experiment. Participants then played the repeated TG with 4 partner types in a 2 × 2 design: partners who were either ingroup or outgroup and either reciprocated frequently (trustworthy) or infrequently (untrustworthy). When playing the repeated Trust Game, the participants remained biased in their investment behavior by investing more trustworthy outgroup partners compared to trustworthy ingroup partners. However, this tendency to invest more with the outgroup compared to the ingroup was not present with untrustworthy partners. Participants’ initial trust bias remained in a positive context with trustworthy partners, however these biases disappeared in a negative context with untrustworthy partners. In other words, participants learned more with the untrustworthy partners in terms of shedding their biases. This study found an initial pro-outgroup trust bias, counter to what most studies find (Balliet et al., 2014). The authors suggest it could be due to participants’ social desirability, given that they were university students who tend to be more conscious about their egalitarianism. However, regardless of the direction of the trust bias at baseline, the fact that participants did modify their behavior as they learned about the trustworthiness of their partners throughout the repeated Trust Game, suggests that the repeated Trust Game could be used to change trust bias.

Given the findings for ingroup-outgroup trust learning, an important area to consider is majority-minority group relations and how trust learning can occur in that dynamic. Discrimination against ethnic minorities is a widespread problem in White-majority Western countries in employment, housing, and medicine (Bertrand and Mullainathan, 2004; Kaas and Manger, 2010; Hoffman et al., 2016). However, several studies have demonstrated that trust is the mediating factor in intergroup contact that results in reducing prejudiced opinions and behavior (Tam et al., 2009; Montoya and Pittinksy, 2011; McKeown and Psaltis, 2017). Therefore, how an existing trust bias can be replaced with positive interpersonal trust *via* (trust) learning, warrants investigation.

In an investigation of racial in- and outgroups, Telga et al. (2018) found that when trustees were fixed to reciprocate on 50% of trials (i.e., maximum uncertainty), White participants in Spain invested more with Black partners than with White partners in a repeated Trust Game. In the next phase of the experiment, participants continued playing as trustors with the same trustees, however the trustees’ reciprocation rates were changed to clear fair and unfair reciprocation rates (80 and 20%, respectively). Participants then played blocks in which all partners were of the same race, including one “outlier” partner who reciprocated differently than the other partners of that race in that block, e.g., one block with 3 unfair White partners and 1 fair White partner. This was done for each race-fairness combination. It was found that participants invested with their White partners based on their individual behavior but tended to invest with Black partners in a generalized manner. More specifically, they invested less with the “outlier” unfair White partner compared to the fair White partners in the same block, and more with the “outlier” fair White partner compared to the unfair White partners in the same block. However, with Black partners, they did not adjust their investment behavior to the outlier partner. In other words, participants were better at individuating White partners compared to Black partners, despite being presented with similar, behavioral evidence. The results suggest that learning to trust may be dependent on the group membership of the partner. However, Vermue et al. (2018) found that it is a combination of the partners’ group membership and their reciprocation that affects learning. Given these related, but somewhat conflicting findings, this warrants further investigation.

Therefore, in the present study, we sought to test how participants’ ethnicity-based trust biases against an ethnic outgroup would change through the repeated Trust Game. As the study was conducted in Germany, Arab people were selected as the ethnic outgroup, as it has been demonstrated that there is significant prejudice against them in German society. For example, White German participants are more likely to shoot and shoot Arab-Muslim targets more quickly than White targets in a shooter game (Essien et al., 2017), Germans expect Muslims to be more aggressive than Christians (Fischer et al., 2007) in the Leipzig authoritarianism study in 2018, 42% of West Germans and 51% of East Germans supported the statement that Muslims should not be allowed to immigrate to Germany (Decker and Brähler, 2018), and in 2019, 871 Islamophobic crimes were reported in Germany, 46 of which resulted in physical injury to the victim (Bayraklı, 2019),^2^ Because of this, we predicted that in a White German sample, participants would initially present with an ingroup trust bias, evaluating their White partners as more trustworthy than Arab partners. We also hypothesized that participants’ biases would change: specifically, that they would learn to trust their partners according to how they behaved in the multi-round Trust Game rather than according to their ethnicity, but that this bias may not be eliminated completely, in line with previous findings (Chang et al., 2010; Fareri et al., 2012, 2015; Vermue et al., 2018).

To investigate the mechanisms of trust learning, we employed reinforcement learning models. This extends the previous research done by Telga et al. (2018) and Vermue et al. (2018) by analyzing the cognitive mechanisms of trust learning. For example, what kind of learning processes are present? Do participants learn differently from losses and gains? Such models can be constructed to test for differences in learning rates according to different aspects of the learning task, including a trial outcome (rewarded vs. unrewarded outcome, a loss outcome vs. a gain outcome), as well as the partner type (ingroup vs. outgroup, fair vs. unfair). Based on previous studies using reinforcement learning models (Fareri et al., 2012, 2015; Lefebvre et al., 2017; Palminteri et al., 2017), we expected participants to learn differently from losses and gains, and that these could vary by the partner’s ethnicity.

With respect to the effectiveness of learning, contact intervention studies have shown that, because of contact, people generalize their changes in prejudice to new members of the outgroup (Greenwald and Pettigrew, 2014). Additionally, learning from one outgroup member can be generalized to other members of that group (Hackel et al., 2022). As such, we expected that when participants reduced their trust bias from learning with specific outgroup members, it would transfer to other members of the same outgroup(close transfer), but not necessarily to members of a different outgroup (distant transfer).

Lastly, we consider the role of implicit bias toward a minority outgroup and how this may relate to general trust bias and trust learning. Implicit attitudes or biases are defined as a favorable or unfavorable feeling, thought, or action toward social objects, of which the person is not aware (Greenwald and Banaji, 1995), which is most widely measured with the implicit association task (IAT) the past 20 years (Greenwald et al., 1998). Some studies have shown significant correlations between implicit racial biases and discriminatory behavior (Senholzi et al., 2015; Essien et al., 2017), including general behavioral trust (Stanley et al., 2011), whereas others show no correlation (Gawronski, 2002; Hofmann et al., 2005). With respect to trust, studies have also shown that trust toward an outgroup and implicit attitudes toward the same outgroup are not related (Tam et al., 2009; Kenworthy et al., 2016). More recent studies question the validity of the IAT entirely (Schimmack, 2021a,b). However, given the past correlations between implicit biases and discriminatory behavior (including behavioral general trust), we included this as an inquiry in our study. To assess the relationship of implicit bias with general trust bias, we included a German-Arab version of the IAT. Half of the participants were given the IAT before the repeated Trust Game, and the other half after the repeated Trust Game. We hypothesized participants who completed the IAT before the repeated Trust Game would demonstrate higher implicit bias than those who completed it after.

In sum, the following hypotheses were tested: that a White, German sample displays an ingroup ethnicity bias, trusting White partners more than Arab partners and that this bias should be significantly changed by playing the repeated Trust Game. In terms of *how* this would change, we predicted the best-fit reinforcement learning model would have separate learning rates for losses and gains and would be affected by the partners’ ethnicity. Additionally, we predicted that this reduced bias would transfer to new members of the ethnic outgroup, but not to members of a different ethnic outgroup. Lastly, we tested for the presence of implicit bias (measured by the IAT) and if this would also be affected by playing the repeated TG.

## Methods

All data, code, and experiment materials including stimuli and participant instructions can be found here: https://osf.io/qzafb/?view_only=af4baf6ce5bc4748ba6704ccb185dc48. The study was not pre-registered; however, sensitivity analyses are provided.

### Participants

A total of 80 participants were tested. Only non-colorblind participants between the ages of 18 and 40 with native or near-native fluency in German were recruited. Two participants were excluded for invalid task behavior (pressing the same button for the duration of the task). Of the remaining participants, we excluded the 5 participants who identified with non-White ethnicities. This resulted in 73 participants (35 male, 38 female), *M_age_* = 28.3, *SD_age_* = 5.6. All participants gave written informed consent for this study, as approved by the ethics committee of Humboldt-Universität zu Berlin.

### Tasks and procedure

Participants performed three tasks: to assess trust bias and trust learning hypotheses, participants rated the trustworthiness of White and Arab partners and then played a modified version of the multi-round Trust Game with the same partners (Berg et al., 1995; Phan et al., 2010). To assess implicit bias, they performed the Implicit Association Test (IAT),^3^ modified for German- and Arab-sounding names (Greenwald et al., 1998; Gawronski, 2002). To assess the transfer of reduced biases to new outgroup members, participants rated the trustworthiness of new, unknown White, Turkish, and Arab faces.

#### Repeated Trust Game

To assess if participants had an ethnicity trust bias, participants were first shown pictures of their partners (6 White and 6 Arab) for the multi-round Trust Game and rated their trustworthiness on a Likert scale from 1 to 7 (1-not at all trustworthy, 7-very trustworthy). Each face was presented for 1 s and participants were told to answer within 3 s. The partners were represented by White-Dutch and Moroccan men from the Radboud face database (Langner et al., 2010).^4^

**Figure 1:**
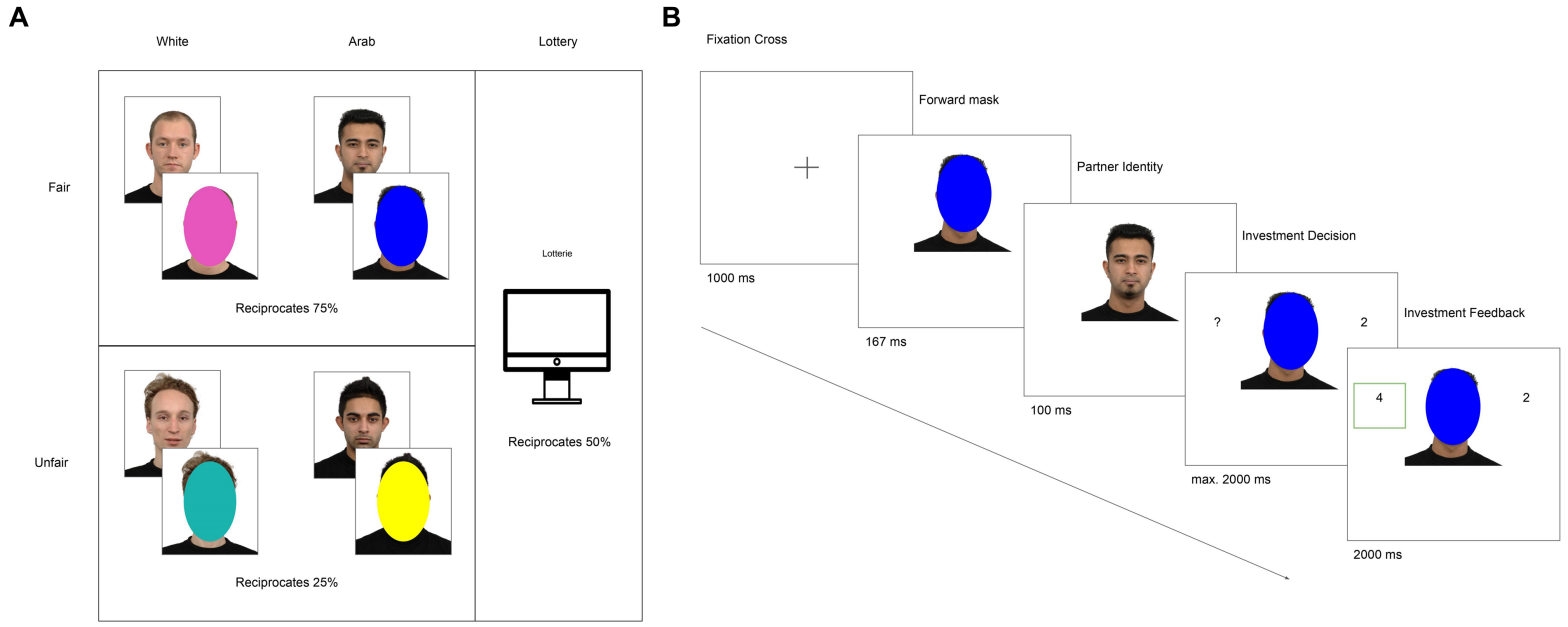
**(A)** Conditions of the interactive Trust Game. **(B)** Exemplar trial showing the interaction with a partner and possible outcomes. Facial images reproduced with permission from Radboud Faces Database (Langner et al., 2010).

To assess how participants learn during the Trust Game as a function of their partners’ behavior and ethnicity, participants played a modified version of the Trust Game (Camerer and Weigelt, 1988; Berg et al., 1995) in a 2 × 2 design with fair-Arab, fair-White, unfair-Arab, and unfair-White partners. Fair partners reciprocated on 75% of the trials and unfair partners on 25% of the trials (Figure 1A). There was also a lottery condition labeled “Lotterie” in which participants received reciprocation on 50% of the trials.

On each trial (Figure 1B), participants had a 4-Euro endowment, which they could either (1) invest entirely with their partner, or (2) keep 2 Euro and give their partner 2 Euro, ending the trial. If the participant chose to invest entirely, the experimenter doubled the investment and the partner received 8 Euros. The trial ended when the partner then either (1) reciprocated 50% of the investment (4 Euro), or (2) defected and kept the full investment. To facilitate learning, on each trial, participants saw their partners’ face for 1 s before it was covered by a colored oval (Phan et al., 2010). They were told each color represented a partner type, who either reciprocated >50% or <50% of the time. Each color (pink, yellow, teal, and blue) was matched a particular condition of the experiment (e.g., unfair-White partners) to help participants generalize the behavior of individual partners to a group category. To further facilitate learning, on each trial participants chose not to invest, they received counterfactual information about their partner’s behavior on that trial, i.e., if their partner would have reciprocated or defected.

To convince participants their partners were real, participants were told that their partners had previously participated in the same experiment as trustees and that their behavior had been recorded (Sripada et al., 2009; Phan et al., 2010). Participants played 3 blocks of 60 trials each (12 trials × 5 conditions) and received feedback about their performance in each block. The trials were pseudorandomized across each block (see Supplementary Information for details). Participants received their average money won from block 3 in addition to their participation money (12 Euros/h). Lastly, participants rated the trustworthiness of their partners post-TG so that changes in their perception of their partners’ trustworthiness could be assessed.

#### Implicit association test

The Implicit Association Test (IAT), as applied to race or ethnicity, measures the speed and accuracy with which participants associate an ethnic or racial group with negative or positive concepts. A modified version of the IAT with German- and Arab-sounding names was used (Greenwald et al., 1998; Gawronski, 2002). See Supplementary Information for stimuli.

#### Trust bias transfer task

To test if participants’ changes in trustworthiness bias would extend to new in- and outgroup members, participants were asked to rate the trustworthiness of 147 new faces on a 7-point Likert scale (1- not at all trustworthy, 7-very trustworthy). The images included the 18 remaining faces from the Radboud dataset which were not assigned to that participant in the repeated TG (9 Moroccan, 9 Dutch-White), as well as 69 images of Turkish faces from the Bogazici database (Saribay et al., 2018) and 60 images of White North Americans from the Chicago face database (Ma et al., 2015; see Supplementary Information for further details). Participants were told they had only 3 s to answer to motivate them to answer quickly. All images were presented in size 600 × 667 pixels with a width to height ratio of 1.11.

## Behavioral analyses

All linear mixed models were carried out using lme4 (Bates et al., 2015) and lmerTest (Kuznetsova et al., 2017) packages in R (R Core Team, 2013), and confidence intervals for the coefficients were calculated using the *confint* function. For *t*-tests, all *p*-values were Bonferroni-Holm corrected for multiple comparisons (Holm, 1979), and as per Lakens (2013), Hedges *g*’ effect sizes (bias-corrected Cohen’s *d*) were calculated. Corresponding confidence intervals for the effect sizes were calculated using ESCI (Exploratory Software for Confidence Intervals; Cumming and Calin-Jageman, 2017) in *Jamovi* (The Jamovi Project, 2021).

### Repeated Trust Game

#### Trustworthiness ratings, pre vs. post TG

To assess our main hypothesis that trust bias (1) exists and (2) would change from repeated interaction in the TG, a multi-level linear model was conducted on the change in trustworthiness ratings, with fixed effects for partner ethnicity (Arab, White), partner fairness (fair, unfair) and their interaction. A random slope of this interaction was included for participants, as well as a random intercept of the partner’s identity. Dependent *t*-tests were conducted to assess: the change in ratings in each category, baseline trustworthiness bias, and post-Trust Game bias. *p*-values were Bonferroni-Holm corrected for multiple comparisons (Holm, 1979).

#### Sensitivity analysis trustworthiness ratings, pre vs. post TG

Given that our main hypothesis was that participants change their trust bias, we conducted a sensitivity analysis for the interaction effect of ethnicity and partner type on changing trustworthiness ratings. We set an *a priori* target sample size of *n* = 80. A power calculation was not done because of the novelty of the paradigm and basing sample sizes on published values could have led to biased sample sizes. To address statistical power, we performed sensitivity analyses with our final sample of 73 participants for the main findings of participants trustworthiness ratings with their partners in the repeated Trust Game. For the linear mixed model, we used the *mixedpower* package in R (Kumle et al., 2021) and sensitivity was estimated for the interaction effects only (see Supplementary Information – Sensitivity Analyses). For *t*-tests, sensitivity was analyzed using the *pwr* package in R (Champley, 2020).

#### Investment decisions

In further support of our main hypothesis, to assess how participants’ investment behavior changed over time according to their partners’ behavior, a mixed effects logistic regression was conducted. The fixed effects were partner ethnicity, partner fairness, and experiment block (1–3), and their interactions. A random intercept was included for the participants.

### IAT and order effects

To address hypothesis participants would have an implicit ethnic bias, and if this would be reduced by playing the repeated TG first, IAT scores were calculated according to Greenwald et al. (2003) and compared to benchmark values from project implicit.^5^ A pre-TG trustworthiness bias score was calculated for each participant by subtracting their mean trustworthiness ratings for their Arab partners from their mean trustworthiness ratings for their White partners. An independent *t*-test was conducted to assess if participants who performed the TG first had a different IAT score than those who performed it second.

### Trust bias transfer task

To assess if participants showed a trust bias with new partners, we assessed if participants rated new faces’ trustworthiness differently based on their ethnicity in 2 models: (1) White vs. non-White, and (2) White (ingroup) vs. Arab (targeted outgroup, close transfer) vs. Turkish (non-targeted outgroup, distant transfer). Linear mixed models with fixed effects for partner ethnicity and ethnicity representativeness, and random effects of partner and participant identity were used. The calculation of ethnicity representativeness is explained in the Supplementary Information. Trials for which participants were too slow (>3,000 ms) or too fast (<50 ms) were excluded from the analysis.

## Reinforcement learning models

Computational reinforcement learning models were used to assess how participants learned in the TG, and how this relates to changing trust biases. We first assessed which learning mechanism best fit our participants: learning from reward or outcome (loss/gain), correctness, simple learning, or no learning. We hypothesized that participants would learn differently from losses and gains based on previous research (Chang et al., 2010; Fareri et al., 2012, 2015). After establishing the mechanism, we took the winning model and applied it to the two ethnicities and two fairness types to compare if participants learned differently from partners based on their ethnicity or their behavioral fairness.

### Models

The models were slightly modified from a traditional *Q*-learning model where the prediction error is calculated by the expected outcome subtracted by the actual outcome. Instead, we modeled participants’ expected probability of their partner reciprocating (*ep*) and then converted it to an expected value of that partner (*ev*) (Fareri et al., 2012, 2015). Specifically, the participants’ expected value, *ev,* of investing with a particular partner type, *i*, is calculated by the expected probability of that partner reciprocating (*ep*), multiplied by the potential reward associated with investing, which was 4 Euros in our experiment:


$$ev_{i}\left(t\right)=ep_{i}\left(t\right)*4$$

The *ep* values were initialized to the participants’ average trustworthiness ratings (and normalized on a scale of 0–1) for that partner type to capture their pre-existing trustworthiness perception of their partners. They were updated *via* prediction error, pe , based on the Rescorla-Wagner prediction error learning equation (Rescorla and Wagner, 1972; Sutton and Barto, 1998),


$$pe\left(t\right)=\gamma -ep_{i}$$

where γ=1 when the partner reciprocates and γ=0 when the partner defects. Importantly, this was calculated the same way on all trials because participants saw their partners’ decision to reciprocate or defect on every trial.

The expected probability of the partner reciprocating is then updated by the prior probability plus the prediction error, which is multiplied by the learning rate, α , as follows:


$$ep_{i}\left(t+1\right)=ep_{i}+\alpha *pe\left(t\right)$$

Participants’ probability of investing on a given trial was then calculated using the Softmax function,


$$P_{invest}=\frac{e^{\frac{ev\left(t\right)}{\beta }}}{e^{\frac{ev\left(t\right)}{\beta }}+e^{\frac{2}{\beta }}}$$

where β is the inverse free temperature parameter and represents participants’ tendency to exploit the currently highest *ev* or explore different options. Here, *ev*(t) is the expected value (in Euro) of investing with a particular partner on a given trial. The expected value of keeping is 2 Euro. The *P_invest_* values were initialized to the participants’ average trustworthiness ratings (and normalized on a scale of 0–1) for that partner type to capture their pre-existing trustworthiness perception of their partners.

#### Models assessing different mechanisms of learning

##### L2G2 (loss × 2, gain × 2)

**Figure 2:**
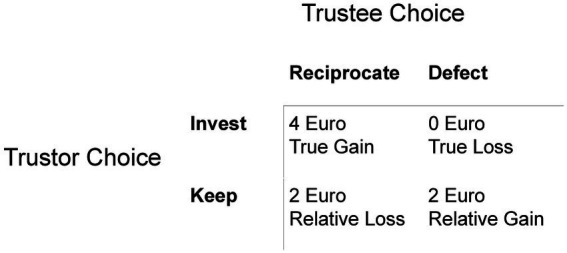
Possible repeated TG trial outcomes.

This is the “full” model which includes 4 learning rates, one for each possible scenario in the Trust Game as depicted in Figure 2. This model assumes that participants learn differently by monetary outcome and correctness about their partners’ choices.

##### LGK (loss-gain-keep)

This model has 3 learning rates and assumes that learning depends solely on the monetary outcome. There is one learning rate for “true” gains, one for “true” losses, and one for keep trials.

##### LG (loss-gain) correctness

This model has 2 learning rates and assumes that participants learn differently from losses and gains, which constitutes making the “correct” choice. The loss learning rate includes both true and relative losses, and the gains learning rate includes both true and relative gains (Figure 2).

##### Reputation

The reputation model assumes that participants learn from their partners’ behavior and not from the monetary outcome. There is one learning rate when a partner reciprocates or would have reciprocated, and one learning rate when a partner defects or would have defected.

##### Simple learn

This model contains one learning rate and assumes that the learning rate does not differ based on partner response or monetary outcome.

##### No learning optimal

The model contains no learning rate and assumes participants invest at the rate that each partner reciprocates, e.g., a non-learning optimal player. Specifically, an optimal player’s expected probability (*ep*) of the partner reciprocating is equal to their reciprocation rate, 0.75 and 0.25 for fair and unfair players, respectively.

##### No learning bias

This model contains no learning rate and assumes participants invest according to their initial trustworthiness impressions of their partners in each partner category and does not update their perception at all. This model captures a “biased non-learner.”

#### Parameter estimation

Following a similar procedure by (Fareri et al., 2012, 2015), we used the negative log-likelihood on each trial to find the optimal parameters. The log-likelihood was calculated as follows:


$$LL=\sum_{t=1}^{n}-\log\left(P_{i,c}\left(t\right)\right)$$

where *i* represents the partner category, *c* indicates the participant’s choice to invest or keep on trial, *t*, and *n* is the total number of trials.

The parameter values for each model were estimated for each participant by passing the negative log-likelihood to Matlab’s *fmincon*, acquiring the maximum likelihood by minimizing the negative log-likelihood. For each participant, each model was run 50 times with different parameter starting values using the *rmsearch* function in Matlab to avoid parameter estimates coming from local minima. The ranges for the free parameters were 0 < β < Infinity and 0 < α <1.

#### Model fitting and comparison

Models were compared using the Bayesian Information Criterion, BIC (Schwarz, 1978), an assessment of model fit which rewards fit but punishes complexity by the number of parameters included. The BIC was calculated for each model, for each participant, for each block (1–3 and composite) as follows:


$$BIC=2*LL+\left(ntrials\right)$$

where LL is the negative log-likelihood and *k* is the number of free parameters estimated in the model. The models were compared by mean BIC across participants and by the number of participants for which each model had the lowest BIC.

## Results

### Repeated Trust Game: pre–post trust ratings

#### Baseline trust bias

**Figure 3:**
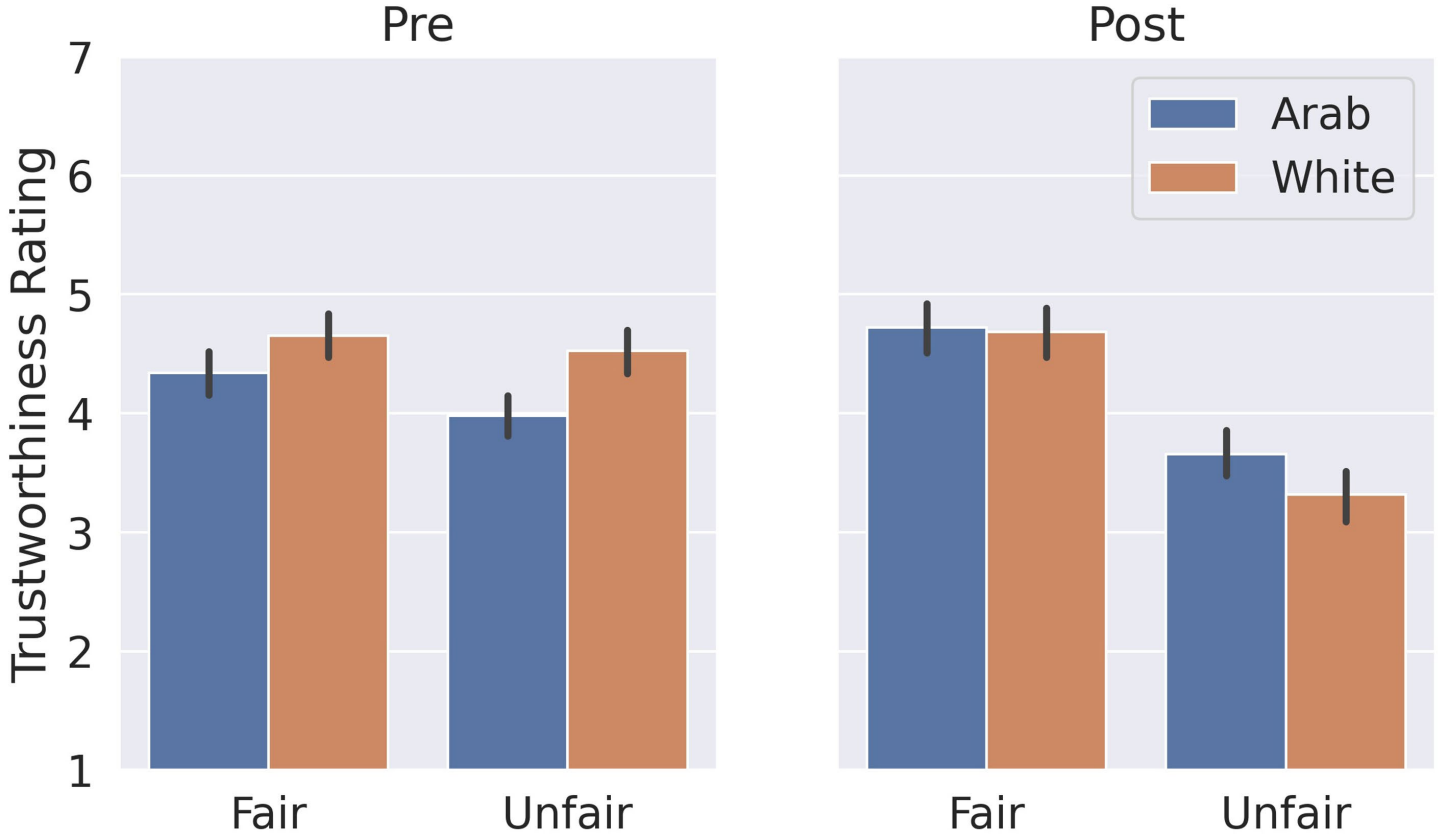
Mean trustworthiness ratings for each partner category at both pre-TG and post-TG interaction time points. The error bars represent 95% confidence intervals.

Participants showed a trust bias prior to the repeated TG (Figure 3), rating White partners (*M* = 4.59, *SD* = 1.39) as significantly more trustworthy than Arab partners (*M* = 4.16, *SD* = 1.39), *t*(72) = 3.19, *p* = 0.013, *Hedges’ g* = 0.46, 95% CI [0.18, 0.76], confirming our hypothesis of baseline ethnic trust bias.

#### Changes in trust and trust bias

The model revealed no significant interaction of partner ethnicity and partner fairness on change in trustworthiness ratings, *β* = −0.38, 95% CI [−0.83, −0.07], SE = 0.23, *t*(72) = −1.7, *p* = 0.10, a significant main effect of partner fairness, *β* = 1.35 95% CI [0.95, 1.75], SE = 0.20, *t*(72) = 6.64, *p* = 4.9e-9, of partner ethnicity, *β* = 0.49, 95% CI [0.12, 0.86], SE = 0.19, *t*(72) = 2.60, *p* = 0.01 and of the participants’ ratings at time pre, *β* = 0.27, 95% CI [0.19, 0.35], SE = 0.04, *t*(72) = 7.2, *p* = 1.47e-12. The largest effect was for partner fairness, indicating it played the largest role of all factors (fairness, ethnicity, and participants’ initial trustworthiness impressions) in updating their perception of their partners’ trustworthiness.

When considering changes for each partner type, participants significantly *decreased* their trustworthiness perception for unfairly behaving White partners (Figure 3), *t*(72) = −7.33, *p* = 1.90e-9, *Hedges’ g* = −1.01 95% CI [−1.32, −0.73] with a large effect. The changes for other partners were not statistically significant. However, there was a trend for participants to increase their trustworthiness perception for fair Arab partners, *t*(72) = 2.59, *p* = 0.058, *Hedges’ g* = 0.30 95% CI [0.07, 0.54] (see Supplementary Table S3 for means and standard deviations and Supplementary Table S4 for correlations).

At the end of the Trust Game, participants rated their fair partners as significantly more trustworthy than their unfair partners after the Trust Game *t*(72) = −7.53, *p* = 1.151e-10, *Hedges’ g* = 1.32 95% CI [0.96, 1.72] demonstrating their trustworthiness perception corresponded to the partners’ behavior. Moreover, participants no longer rated their partners differently based on their ethnicity, neither for fair partners, White vs. Arab, *t*(72) = −0.184, *p* = 0.854, *Hedges’ g* = −0.03 95% CI [−0.34, 0.28], (2) nor for unfair partners, White vs. Arab, *t*(72) = −1.80, *p* = 0.076, *Hedges’ g* = −0.28 95% CI [−0.60, 0.03], indicating their trust bias was changed.

Note that there was a statistically significant difference in participants’ baseline (pre-TG) trustworthiness ratings of fair Arab (*M* = 4.34, *SD* = 1.13) and unfair Arab partners (*M* = 3.98, *SD* = 1.01), *t*(72) = −3.28, *p* = 0.013 (Holm-corrected for multiple comparisons), *Hedges’ g* = −0.33, 95% CI [−0.55, −0.13]. This is likely due to the images not being perfectly counterbalanced (see Supplementary Information for details on image selection). However, we addressed this by including the pre-ratings as a covariate in the regression model and by including the pre-ratings in the reinforcement learning models. Therefore, our analyses take into account participants’ pre-ratings in how they learned over time.

#### Sensitivity analysis – change in trust bias

The minimum detectable effect size of a partner ethnicity-fairness interaction on pre-post trustworthiness ratings would be −0.65 (see Supplementary Information for details) with 73 participants and 80% power. Therefore, our sample size was too small to detect this interaction. The minimum detectable effect size for changes in trustworthiness ratings with each partner type would be 0.30, using dependent two-tailed t-tests, 80% power, 73 participants, and alpha = 0.05. The change in trustworthiness perception for unfair white partners was −7.3, and for fair Arab partners 0.30, indicating our study was sufficiently powered for the change in the former, but not the latter (although the latter is close to the minimum effect).

Taken together, the experiment was sufficiently powered to detect changes in trustworthiness perception for the unfair White partners, and that while there may be a trend to change trustworthiness for fair Arab partners, it did not reach statistical significance (therefore an interaction effect was also not detected).

### Repeated Trust Game investment behavior

**Figure 4:**
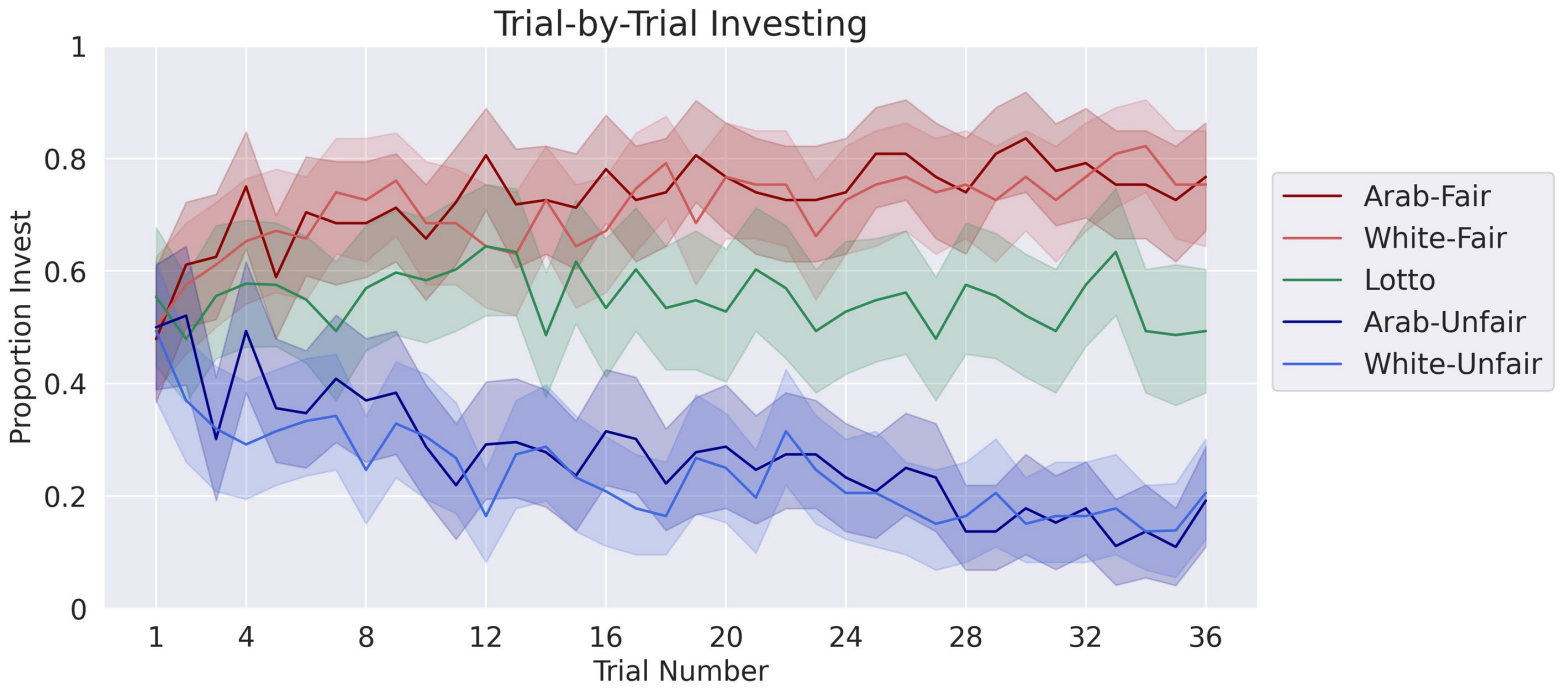
Participants’ proportion invested in the Trust Game with each partner type over time. The shaded areas represent 95% confidence intervals.

To assess how participants trusted/invested with their partners throughout the TG, the model revealed a significant interaction of block and partner fairness, *β* = 0.68, 95% CI [0.52, 0.84], z = 8.50, *p* < 0.001, as well as main effects for partner ethnicity, *β* = 0.43, 95% CI [0.10, 0.75], z = 2.6, *p* = 0.01, partner fairness, *β* = 0.83 95% CI [0.51, 1.5], z = 5, *p* < 0.001, and experiment block, *β* = −0.42, 95% CI [−0.53, −0.30], *z* = −7.1, *p* < 0.001. There was no significant interaction of partner ethnicity and block, nor for partner ethnicity and fairness. This indicates that participants changed their investment behavior over time according to their partners’ fairness (Figure 4). See Supplementary Table S2 for investment means and standard deviations for each condition.

### IAT and order effects

Participants demonstrated a medium-strength implicit pro-White bias, favoring White partners over Arab partners on the IAT (IAT score: *M* = 0.41, *SD* = 0.53). The strength of this effect is determined according to Project Implicit, which qualifies the IAT scores as follows: 0.15 = slight pro-White bias, 0.35 = moderate pro-White bias, 0.65 = strong pro-White bias. Participants who completed the IAT first (*M* = 0.36, *SD* = 0.54) did not score significantly differently on the IAT than those who did it second (*M* = 0.46, *SD* = 0.53), *t*(71) = −0.86, *p* = 0.391, *Hedges’ g* = 0.20, 95% CI [−0.26, 0.67], indicating that playing the repeated TG did not affect IAT scores.

Participants who completed the multi-round Trust Game first (*M* = 0.53, *SD* = 1.04) did not have a significantly different trustworthiness bias score than those who completed it after the IAT as the second task (*M* = 0.33, *SD* = 1.25), *t*(71) = −0.74, *p* = 0.462, *Hedges’ g =* 0.18, 95% CI [−0.28, 0.65], indicating that the TG did not have an effect on IAT scores.

### Trust bias transfer task

The model comparing White vs. non-White partners found no effect of ethnicity on the trustworthiness ratings, *β* = 0.03, 95% CI [−0.17, 0.22], SE = 0.10, *t*(161) = 0.255, *p* = 0.799, indicating that participants’ reduced biases extended to new outgroup faces (see Supplementary Table S5 for full model). In the model which tested for 3 ethnicities (White, Arab, and Turkish), there was no significant difference in trustworthiness ratings of Turkish partners compared to White partners, *β* = 0.09, 95% CI [−0.13 0.31], SE = 0.11, *t*(198) = 0.81, *p* = 0.418, nor for Arab partners compared to White partners, *β* = −0.23, 95% CI [−0.51 0.06], SE = 0.15, *t*(201) = −1.5, *p* = 0.124. There was also no significant effect of the ethnicity-typicality covariate, *β* = −0.53, 95% CI [−1.5 0.41], SE =0.48, *t* = −1.1, *p* = 0.271 (see Supplementary Table S6 for full model). These results seem to indicate that participants’ reduced biases extend to new members of the Arab outgroup, and possibly toward the new outgroup of Turkish faces. However, we did not measure for a baseline bias toward Turkish faces, and therefore this result alone is inconclusive.

#### New outgroup

To address this, we conducted a follow-up online study to establish if a similar sample would show a trust bias favoring White partners over Turkish partners. We tested 101 participants. Eighteen were excluded to match our lab sample: 4 were too slow (>3,000 ms) or too fast (<50 ms) on 15% or more of their trials and 14 participants did not identify with White ethnicities. Trials that were too long (>3,000 ms) or too quick (<50 ms) were excluded. Participants (*n* = 83) rated the same images that were used in the lab (60 White-North American, 69 Turkish, 15 Moroccan, 15 White-Dutch) without a Trust Game intervention. A linear mixed model with partner ethnicity (three: White, Turkish, Arab) as a fixed effect, ethnicity typicality as a covariate, and with participant and partner identity as random effects (intercepts and slopes) on participants’ ratings was conducted.

Participants did not rate their Turkish (*M* = 3.81, *SD* = 1.53) and White partners (*M* = 3.75, *SD* = 1.55) significantly differently on trustworthiness *β* = −0.05, 95% CI [−0.27 0.16], SE =0.11, *t*(210) = −0.52, *p* = 0.607. However, Arab partners were rated as significantly less trustworthy than White partners, *β* = −0.28, 95% CI [−0.54–0.01], SE =0.14, *t*(195) = −2.1, *p* = 0.041, replicating our previous findings. See Supplementary Table S7 for full model. Therefore, given that we do not observe a baseline trust bias toward Turks in our sample, we cannot conclude if the reduced trust bias from the repeated TG extends to a new outgroup.

### Reinforcement learning models

#### Model fit and winning model selection

The simple learning model had the lowest mean BIC of 136 (Table 1) compared to all other models. Using the guidelines from Raftery (1995), the difference in BIC is only considered informative when it exceeds 2. The difference in mean BIC fit between simple learn and the second best-fitting model, LG, is 13, indicating a meaningful difference.

**Table 1:** Estimated model parameters and BIC.

	NL bias	NL optimal	Simple learn	LG	LGK	L2G2	Reputation
BIC	196.4 (20.49)	151.07 (57.11)	134.14 (50.28)	149.22 (54.87)	156.23 (51.04)	155 (53)	151.39 (50.15)
β	1371.94 (1950.55)*	152.62 (635.32)*	0.62 (0.45)	1.32 (1.20)	1.08 (1.12)	1.09 (0.92)	1.13 (1.61)
α	**–**	**–**	0.16 (0.10)	G: 0.37 (0.24)	G: 0.26 (0.30)	G: 0.31 (0.29)	D: 0.22 (0.23)
				L: 0.09 (0.11)	L: 0.21 (0.24)	L: 0.15 (0.21)	R: 0.19 (0.24)
					K: 0.19 (0.20)	G-rel: 0.39 (0.29)	
						L-rel: 0.13 (0.20)	

Mean (SD) for BIC, beta, and respective alpha values for each model are presented in the table. *For the NL optimal model, there were 4 outliers with *β* values >1900. Excluding these yields, *M* = 2.1, *SD* = 3.6. For the NL bias model, extreme beta values were common, with 38 participants having beta values >1,000.

**Figure 5:**
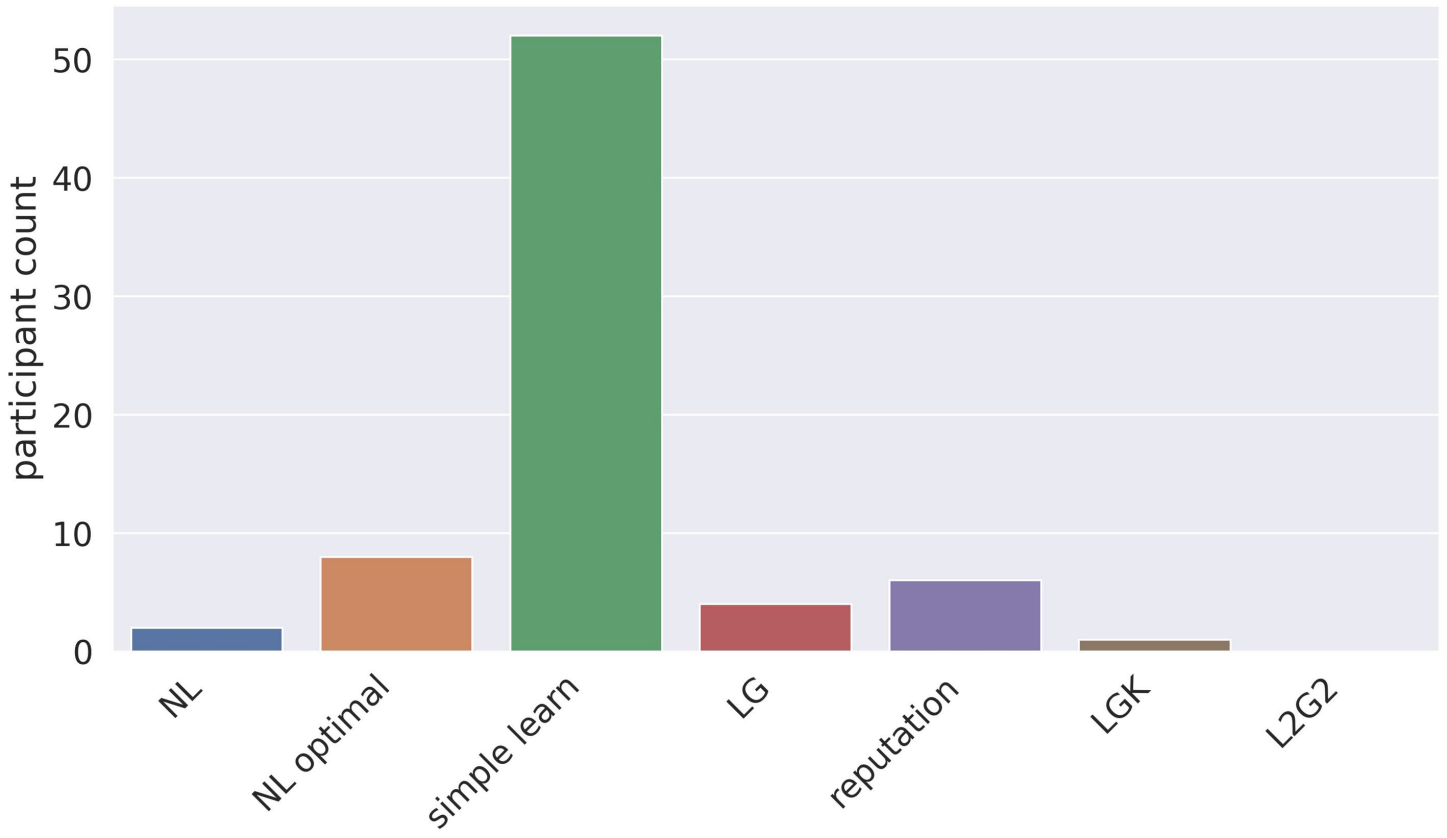
The number of participants for whom each model had the best fit, as calculated by lowest BIC.

Because the mean is sensitive to extreme values, we also analyzed how frequently a model provided the best fit for participants’ behavior. This comparison showed that the simple learn model provided the best fit for the highest number of participants at 52 participants (Figure 5; for comparison by block see Supplementary Figure S1).

### Frequency of best fit for each model

Taken together, the BIC comparisons revealed that the simple learn model had the best fit: its mean BIC was the lowest, and its BIC was the lowest for the greatest number of participants.

#### Model validation

To further confirm if the simple learn model had the best fit, we performed a model validation procedure with simulated data from the top three models: simple learn, reputation, and LG. Specifically, the estimated parameters for each participant were used to create 100 simulations for each subject for each of these three models.

**Figure 6:**
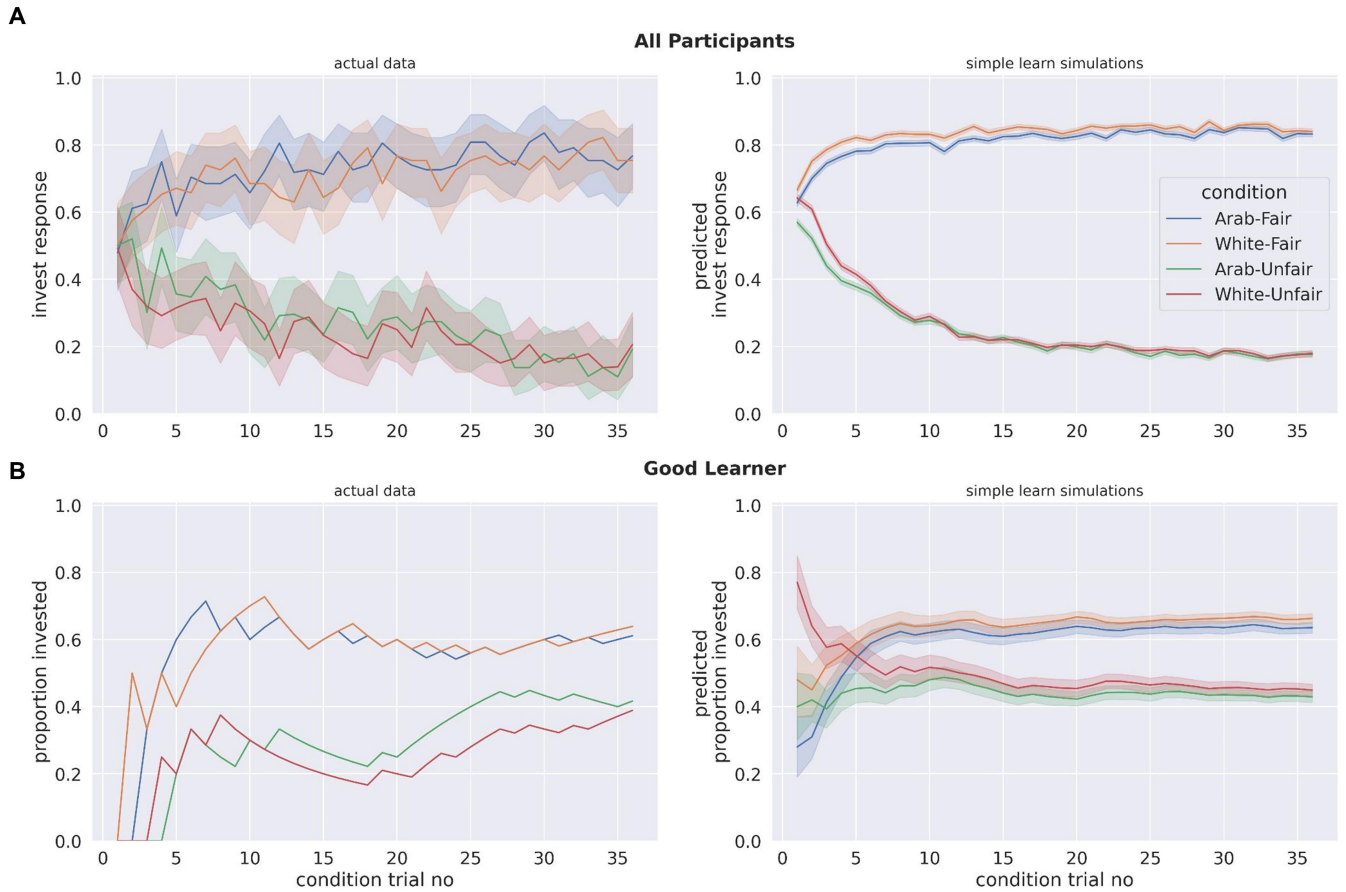
**(A)** A comparison of the participants’ actual investment responses with each partner type in the experiment with invest responses estimated by 100 stimulations for each participant from the simple learn model. For participants’ actual data, the proportion of invest responses made by all participants at a given condition trial were calculated. For simulations, this was calculated the same way, except not only across all participants but all simulations as well. **(B)** An example of a single participant’s actual data vs. simulated data from the simple learn model. A “good” learner was selected as example: someone who learned to invest more with the fair partners than with the unfair partners. In **(A,B)** the shaded areas represent 95% confidence intervals.

Figure 6 shows participants investment behavior compared to the predicted behavior from the model simulations. In Figure 6A, the average across all participants is presented, and in Figure 6B an example for a single participant (who was a good learner) is shown. Both show that investments with partners in early trials are overestimated in the simple learn model, however this was also the case for the reputation and LG models as well (Supplementary Figure S2; simulations for a bad learner and a decent learner are in Supplementary Figure S3). The simple learn model provided the closest fit to participants’ actual behavior, further indicating that it is the best-fitting model for participants’ learning in this study.

#### Simple learn ethnicity and fairness models

After establishing the main learning mechanism (reward, correctness, etc.), we analyzed two follow-up models to test if participants learned differently from their partners based on their ethnicity and fairness. Both models followed the simple learn algorithm in that trial outcomes and correctness all shared the same learning rate (gains, relative gains, losses, relative losses were all under one learning rate), but allowed for different learning rates based on the partner. The simple learn ethnicity model contained 1 learning rate for Arab partners and 1 for White partners. The simple learn fairness model contained 1 learning rate for fair partners and 1 for unfair partners. Mean BIC values across participants were compared, as was the number of participants for whom each model had the lowest BIC value.

Both models performed worse than the simple learn model. It still had the lowest mean BIC value for each block and for the composite experiment (Supplementary Table S9 and Supplementary Figure S4A), by 10 BIC points, constituting a significant difference (Schwarz, 1978). The simple learn model additionally had the lowest BIC for all participants except one (Supplementary Figure S4B).

## Discussion

This study sought to examine if ethnicity-based trust biases could be reduced by allowing participants to interact with fictitious partners of different ethnicities and learn their actual trustworthiness through a repeated Trust Game. The (White) participants were initially biased, believing that other White partners were more trustworthy than Arab partners, replicating previous findings that participants evaluate ingroup members to be more trustworthy than outgroup members (meta-analysis: Balliet et al., 2014). We further replicated this finding in a follow-up experiment with 83 new participants. During the multi-round Trust Game, however, participants invested with their partners according to their behavior and not their ethnicity, specifically investing more often with fair partners than with unfair partners, supporting previous findings (Telga et al., 2018; Vermue et al., 2018). At the end of the multi-round Trust Game, participants’ ethnicity-driven trust biases, in the form of rating their partners’ trustworthiness, disappeared. In becoming unbiased, participants significantly decreased their trustworthiness perception of unfair White partners. Additionally, when given new White and Arab partners to evaluate, participants remained unbiased in their trustworthiness evaluations, indicating that they generalized what they learned to new individuals. However, as the set of new Arab faces was small (9 faces), this result should be interpreted with caution and would benefit from replication with a larger set of faces.

### Reinforcement learning and reducing ingroup trust bias

In terms of *how* participants learned during the Trust Game, we hypothesized they would learn differently from positive and negative outcomes (Fareri et al., 2012, 2015; Lefebvre et al., 2017; Palminteri et al., 2017), and that this would be moderated by the partners’ ethnicity. More specifically, we predicted that a reinforcement learning model with one learning rate for gains and one for losses would be the best fit model. Contrary to our expectations, we found robust evidence for the simplest model with one learning rate. Moreover, participants did not learn differently based on their partners’ ethnicity or fairness throughout the task. The finding that one learning rate fit participants’ behavior best, indicates that participants weighted their prediction errors similarly across monetary reward, correctness, and the partner’s ethnicity and behavior. Moreover, participants learned to invest with the correct partners (maximizing their winnings) and in the process, to change their ethnicity-driven trustworthiness bias.

Our results support previous findings that during a multi-round Trust Game, participants do change their trustworthiness perception of their partners. However, according to those previous accounts it is not eliminated entirely (Chang et al., 2010; Fareri et al., 2012, 2015; Vermue et al., 2018). In our study, by contrast, participants’ ethnicity-driven trust biases were eliminated, and this learning process was explained by a simple model in which participants weighted evidence from all outcomes equally.

Telga et al. (2018), which also used the repeated TG for racial in- and outgroups, found that participants initially showed a pro-outgroup bias, investing more with outgroup members than ingroup members. Participants were then able to learn the trustworthiness of their partners, but their partners’ race did affect their learning. Specifically, participants learned the general behavior of the outgroup and ingroup members, and when an ingroup member behaved differently from the rest of the ingroup, participants learned this. However, when an outgroup member behaved differently from the rest of the outgroup, participants did not learn this and invested with them as they did with the rest of the outgroup. Similarly, Vermue et al. (2018) found that participants invested more with outgroup nationalities than with ingroup nationalities, and that this only partially changed after playing the repeated TG.

Our study supports the finding that participants generally learned to invest with their partners according to their behavior and not their group status. However, our study differs in that we found a significant pro-ingroup trust bias, that participants learned from all partners similarly (specifically their prediction errors were weighed equally with one learning rate) and that their pro-ingroup trust bias was eliminated by playing the Trust Game.

In general, the results of Telga et al. (2018) and Vermue et al. (2018) align with results outside of an ingroup-outgroup context, showing “partial learning,” i.e., participants learning to update their perceptions to some extent, but not shedding their biases completely (Chang et al., 2010; Fareri et al., 2012, 2015). It was precisely our goal to adjust the paradigm to maximize learning, so participants would reduce their biases, and we therefore outline the reasons why it was successful in comparison to other studies.

There are several features of the study which might explain our results and why they differ from other experiments: First, we used a version of the Trust Game which gave participants counterfactual information about their partners’ behavior, allowing them to learn about their partners on every trial, whereas in the aforementioned studies, participants only learned from their partners when they chose to invest. Studies have shown that participants do use counterfactual information in learning and that learning rates for factual and counterfactual information do not differ significantly (Fischer and Ullsperger, 2013; Lefebvre et al., 2017; Palminteri et al., 2017). This may explain why our participants learned quickly and why a relatively simple model explains our participants’ learning compared to other studies.

Another crucial point is that studies that included fair/good and unfair/bad partners in the form of high and low reciprocation rates, respectively, have shown a stronger result in reducing prior biases (Delgado et al., 2005; Chang et al., 2010; Telga et al., 2018), compared to those that included the maximally uncertain reciprocation rate of 50% (Fareri et al., 2012, 2015). Our experiment also used reciprocation rates of 75 and 25% creating a clear distinction of fair and unfair behavior, similar to the studies that showed bias reduction. If reciprocation or reward rates are maximally uncertain, the evidence about this partner’s behavior is ambiguous, and may lead participants to rely more on their biases. This is a critical task design point to make sure that participants do reduce their biases over the task, even if they do not do so completely.

Additionally, participants in our study were investing in a loss-frame. We used this structure so participants would be more focused on their partners’ frequency of reciprocation aside from profit. Specifically, in our study, participants start with 4 euro and in the best-case scenario, end up with 4 euro if they invest and their partner reciprocates. In a defection scenario, they end up with 0 Euro, and in a keep scenario, they end up with 2 Euro. However, this differs from the typical TG design in which participants are playing in a gain-frame, in which participants end up with more money than their original endowment from an invest-reciprocate scenario. Despite the loss-frame, we did observe that the investment curves match those from other studies in a which a gain structure is used (Delgado et al., 2005; Chang et al., 2010; Telga et al., 2018), suggesting learning in the repeated TG is similar. However, loss-gain framing can affect participants’ behavior, given people are more sensitive to outcomes framed as losses (Kahneman and Tversky, 1979; Camerer, 2004; Sokol-Hessner et al., 2009). In the (non-repeated) Trust Game specifically, it has been found that participants in a loss frame trusted more often and that decisions (both for trustors and trustees) were less calculative in the loss frame than participants playing in a gain-frame (Evans and van Beest, 2017). The authors further suggest that loss framing has the strongest positive effect on trust when there is a low expected value, either due to low expectations or unfavorable payoffs. Another possible reason that our results differ from existent literature is that participants received knowledge of their partners’ investment behavior on every trial, regardless of investment, allowing learning to occur regardless of the participant’s investment decision or not. In the future, it would be prudent to examine both loss and gain frames, crucially when counterfactual information is present, and if this combination influences participants’ trust learning.

Lastly, the priors about the partners play a role in how participants update their perception of their partners trustworthiness from interacting in the TG. Studies that include explicit manipulations with information about partners’ morality or trustworthiness create stronger priors than studies that rely on implicit information such as facial trustworthiness appearance, emotion expression, or ethnicity, as evidenced by the persistence of those priors in investment behavior (explicit: Delgado et al., 2005; Zarolia et al., 2017; implicit: Chang et al., 2010; Telga et al., 2018; Fujino et al., 2020). Although ethnicity can be used as a proxy for trustworthiness reputation, affecting initial investments (Stanley et al., 2011), this information can be quickly discarded in the face of evidence about trustworthiness. This is what we find and is in part supported by previous findings (Telga et al., 2018). It appears that ethnic biases may not serve as strong priors and are more malleable in the face of information compared to explicit than other forms of biases, such as social closeness (e.g., preferring a friend to a stranger) or explicit information about trustworthiness (Fareri et al., 2012, 2015, respectively).

This may offer an explanation as to why we did not find different learning rates for partners based on their ethnicity; specifically, the ethnicity-driven priors might have been taken over by behavioral evidence. Another explanation is that the colors were salient enough to mask the effect of the facial identity and ethnicity. However, participants rated their partners at the end of the task *without colors*, and their ratings were significantly higher for the fair partners than unfair partners, regardless of ethnicity. This shows that participants paid attention to the facial identities and their associated behavior, and therefore changed their trustworthiness bias by learning the true trustworthiness of those partners.

### Counter-bias exemplars: the “bad” ingroup member

Although participants did not invest with or learn differently from their partners based on their ethnicity, the largest change in participants’ trustworthiness perception was for unfair White partners. This highlights the importance of the unfair ingroup member in reducing group-based biases, as it has been shown that participants’ biases are the product of ingroup favoritism, rather than outgroup derogation (Balliet et al., 2014; Everett et al., 2015; Romano et al., 2017) and that the most effective interventions include negative ingroup exemplars in addition to positive outgroup exemplars (Lai et al., 2014, review: Fitzgerald et al., 2019). Specifically, participants are sensitive to ingroup “betrayal” and will adjust their ingroup favoritism accordingly (Valenzuela and Srivastava, 2012; Mendoza et al., 2014). It has also been shown that participants have enhanced memory of uncooperative ingroup members compared to uncooperative outgroup members (Hechler et al., 2016). These findings, coupled with the tendency for participants to trust ingroup members more initially, point to why participants’ largest change in trustworthiness perception was for the unfair ingroup partners.

### Generalizability of reduced trust bias

Participants’ learning was substantial enough to not only change their trustworthiness biases for their partners in the task, but also to continue to be unbiased when presented with new members of those in and outgroups. However, this “transfer” was only tested with 9 outgroup faces. Despite the small sample, this points to the effectiveness of the task at reducing biases for new members of the target outgroup. To test if this bias reduction can extend to other outgroups, future studies should include two outgroups for which there are significant pre-existing biases.

Additionally, it should be noted that playing the multi-round Trust Game did not produce a significant change in implicit intergroup attitudes, as measured by the implicit association test (IAT). Specifically, there was no difference in IAT scores between participants who took the IAT before the multi-round Trust Game and those who took it after. However, this is not unexpected as previous research indicates that trust and attitudes toward outgroups are mutually dissociable phenomena (Tam et al., 2009; Kenworthy et al., 2016) and that changing implicit attitudes does not result in changing explicit attitudes nor prejudiced behavior (Oswald et al., 2013; Lai et al., 2014). Further, the IAT has been shown to lack construct validity and therefore is questionable what it truly measures (Schimmack, 2021b). Our findings support this dissociation in that implicit biases were unrelated to trust learning and trustworthiness attitudes.

### Limitations

One limitation of the study is how the transfer effect was designed, namely with a small set of images, and without pre-ratings for the second outgroup. In future studies, a larger stimulus set should be used. In terms of testing a second outgroup, we assumed that Turkish and Arab faces would be perceived similarly in photos and used both Turkish and Arab faces to test the transfer effect. We were incorrect in this assumption, which was revealed in a follow-up online study, which showed that participants did have a trustworthiness bias toward Arab faces, but not toward Turkish faces. Therefore, we cannot establish if changing trust bias for one group could also change trust biases for another group, and this warrants further investigation.

Secondly, the strength of this effect should be questioned by testing the effects of this long-term. With contact interventions, the effects seem to last after 1 month or more (Lemmer and Wagner, 2015), however with implicit bias interventions, the effects tend to be short-lived (none last longer than a few days), although they do seem to extend to new ethnic outgroups (Lai et al., 2016). The results found in this study would benefit greatly from additional testing which studies the longevity and generalizability of these effects more extensively.

Additionally, the images used in the trust task were not perfectly balanced across participants, resulting in unfair Arab partners being rated as significantly less trustworthy than fair Arab partners before the start of the task. However, this is accounted for in our analyses in the following ways: the linear mixed model analyzes participants’ post-trustworthiness ratings includes the pre-ratings as a covariate, adjusting for baseline differences. It also includes a random effect for each individual partner picture. Therefore, individual effects of images (such as a particularly untrustworthy or trustworthy face) are taken into account. Additionally, when assessing the learning process, participants’ trustworthiness ratings are used to initialize key variables in the model, therefore also accounting for baseline variations on an individual participant basis.

Lastly, participants’ learning was facilitated by the color which covered the partners’ faces. The colors were chosen somewhat arbitrarily. Although we found no effect of the color on learning (see Supplementary Information), a more parsimonious approach could be to test for likeability of colors and adjust for that beforehand.

## Conclusion

The present study demonstrated that participants who originally perceived their ethnic ingroup to be more trustworthy than an ethnic minority outgroup, became unbiased as a result of playing the multi-round Trust Game. We presented ethnic in- and outgroups as both trustworthy and untrustworthy, leading participants to judge their partners based on their behavior and not biases about their ethnicity. Reinforcement learning models demonstrated that participants learned in an unbiased manner: trial outcomes (losses/gains) and partner types were weighted equally. Importantly, what participants learned extended to new outgroup members, indicating the robustness of participants’ learning.

## Data availability statement

The datasets presented in this study can be found in online repositories. The names of the repository/repositories and accession number(s) can be found at: https://osf.io/qzafb/?view_only=af4baf6ce5bc4748ba6704ccb185dc48.

## Ethics statement

The studies involving human participants were reviewed and approved by ethics committee of Humboldt-Universität zu Berlin. The patients/participants provided their written informed consent to participate in this study. Written informed consent was obtained from the individual(s) for the publication of any potentially identifiable images or data included in this article.

## Author contributions

CD, ID, and HW conceived the design. CD collected the data and wrote the first draft of the manuscript. CD and UT analyzed the data. All authors contributed to manuscript revision, read, and approved the submitted version.

## Funding

This study was supported by the Berlin School of Mind and Brain.

## Conflict of interest

The authors declare that the research was conducted in the absence of any commercial or financial relationships that could be construed as a potential conflict of interest.

## Publisher’s note

All claims expressed in this article are solely those of the authors and do not necessarily represent those of their affiliated organizations, or those of the publisher, the editors and the reviewers. Any product that may be evaluated in this article, or claim that may be made by its manufacturer, is not guaranteed or endorsed by the publisher.

## Footnotes

^1^Note that cooperation and trust are similar constructs and highly correlated (Yamagishi et al., 2013). In the context of economic games measuring trust, such as the trust game, trust and trustworthiness are both operationally defined as costly other-benefiting behavior and they can be seen as special forms of cooperation (Yamagishi et al., 2013).

^2^For a historical perspective, see Lewicki (2018).

^3^To account for task order effects, half of the participants performed the multi-round trust game first, followed by the IAT (*n* = 36, 17 male, 19 female) and the other half of the participants performed the IAT before the multi-round trust game (*n* = 37, 18 male, 19 female). As the last task, participants rated the trustworthiness of 135 unique faces to test if their reduced trustworthiness biases would transfer to new faces. However, we did not test participants immediately to account for the possibility that participants’ biases may return when given a break. As such, before testing for a transfer effect of reduced bias, participants took a 15-min break during which they were given a 5-min, no-screen break followed by a 10-min video about Kayaks. At the end of the experiment, participants provided demographic information, were paid for their participation, and were debriefed on the purpose of the experiment.

^4^Prior to this experiment, an independent sample of participants (*n* = 25) rated these stimuli on attractiveness and ethnicity representativeness. The stimuli were then matched on these parameters and then assigned to the behavioral fairness conditions such that each fairness condition contained attractive and unattractive partners of each ethnicity (see Supplementary Information for further details).

^5^ https://www.projectimplicit.net/
