# Supplementary material for: Ethnic discrimination unlearned: experience in the repeated Trust Game reduces trust bias
Source: Front Psychol. 2023 May 25;14:1139128. doi: 10.3389/fpsyg.2023.1139128 (PMC10249959; doi:10.3389/fpsyg.2023.1139128)
Supplement: Supplementary file 1 [file Data_Sheet_1.docx]

Supplementary Information and Results

# Stimuli

## Trust Game Stimuli

Participants were pseudorandomly assigned 12 partners (6 White, 6 Arab) from the Radboud Face Database (Langner et al, 2010), 6 of whom would behave fairly, and 6 of whom would behave unfairly in the multi-round Trust Game, balanced for ethnicity.

In order to pair the stimuli appropriately, we first had an independent group of participants in Germany (*n* = 25), rate the Radboud faces (20 White-Dutch faces and 18 Arab-Moroccan faces) on trustworthiness, attractiveness (1-7 Likert scales), and on an ethnicity slider-scale of White – Middle-Eastern, with 1 as 100% White and 101 as 100% Middle Eastern. The attractiveness scores were pooled with the attractiveness ratings that came with the Radboud Face database to yield a final attractiveness score for each image.

To avoid using ethnically ambiguous faces, only the faces that were rated on average as >80% White or Middle Eastern were added to the subset. The images within this subset were then matched by attractiveness and ethnicity representativeness ratings using the *optmatch* R package (Hansen and Klopfer, 2006). The final subset contained 15 optimally matched, White-Arab image pairs.

The image pairs were then divided into high, medium, and low attractiveness levels: the 5 image-pairs with the highest attractiveness ratings were labeled as “high” attractiveness, the 5 image-pairs with the lowest attractiveness ratings were labeled as “low” attractiveness, and the 5 image-pairs with attractiveness ratings in the middle were labeled as “medium” attractiveness. For each participant, 1 face pair was drawn randomly from each attractiveness category, such that fair and unfair categories included attractive, medium-attractive, and unattractive faces of each ethnicity. The color was randomly assigned to each category for each participant.

For the practice rounds, the faces used were the White faces from the Radboud Face database that were not selected for the manipulation portion of the experiment; i.e., all participants saw the same faces in the practice round (although fairness assignment varied), and these faces were not included in any experimental rounds for any participant. The colors in the practice round were not used in the experimental rounds.

All images were presented in size 600 x 667 pixels with a width to height ratio of 1.11, with an image resolution of 72 x 72 dpi.

## IAT Stimuli

**SI Table 1. Stimuli used in the Implicit Association Test**

| German Names | Arab Names | Positive Words | Negative Words |
| --- | --- | --- | --- |
| Günther | Hakim | glücklich (happy) | Qual (agony) |
| Matthias | Sharif | Frieden (peace) | verletzt (hurt) |
| Harald | Yousef | Vergnügen (pleasure) | Misserfolg (failure) |
| Stefan | Wahib | prachtvoll (magnificent) | böse (evil) |
| Dieter | Akbar | Liebe (love) | Übel (evil/bad) |
| Wolfgang | Muhsin | Lachen (laughter) | schrecklich (terrible) |
| Volker | Salim | Freude (joy) | grausam (cruel) |
| Michael | Karim | wundervoll (wonderful) | scheußlich (horrible) |
| Konrad | Habib |  |  |
| Eberhard | Ashraf |  |  |

German names were taken from Gawronski (2002), Arab names were taken from the American IAT with Arab-Muslims vs. Other People (Nosek et al, 2007; for an example, see: <https://implicit.harvard.edu/implicit/selectatest.html>) and the positive and negative words were taken from the German IAT (translated from Nosek et al, 2007 by Prof. Konrad Schnabel; for an example, see: <https://implicit.harvard.edu/implicit/germany/takeatest.html>).

## Transfer Task Stimuli

The images selected from the Chicago face database (Ma, Correll, & Wittembrink, 2015) were limited to the age range 20-35 and had a rating of 80% White or greater (i.e., 80 percent of the participants in their study rated these images as “White”). The images selected from the Bogazici face database (Saribay et al., 2018) were limited to the age range 18-28. Participants in the Bogazici face database rated the faces for how Turkish they appeared (“Turkishness”) on a 1-5 Likert scale (Not at all Turkish - Very Turkish). The Likert scale was converted to a scale of 0-1 to be comparable with the ethnicity ratings for the images in the Chicago face database. Once converted, the images were selected for 75% Turkishness, as a cut-off of 80% yielded too few images. Again, the images were presented in size 600 x 667 pixels with a width to height ratio of 1.11. For the Chicago faces, the resolution was 240 x 240 dpi; for the Bogazici faces, it was 508 x 508 dpi.

# Behavioral Results

## Investment Behavior

**SI Table 2. Participants' mean investments by block and partner.**

| Block | 1 | 2 | 3 | Composite |
| --- | --- | --- | --- | --- |
| Lotto | 0.57 (0.50) | 0.56 (0.50) | 0.53 (0.50) | 0.55 (0.50) |
| Arab-Fair | 0.67 (0.47) | 0.74 (0.44) | 0.78 (0.42) | 0.73 (0.44) |
| Arab-Unfair | 0.37 (0.48) | 0.27 (0.44) | 0.17 (0.38) | 0.27 (0.44) |
| White-Fair | 0.66 (0.47) | 0.71 (0.45) | 0.76 (0.43) | 0.71 (0.45) |
| White-Unfair | 0.31 (0.47) | 0.24 (0.42) | 0.17 (0.38) | 0.24 (0.43) |

M (SD). Note these are raw means & SDs, not first aggregated by participant.

## Trustworthiness Perception Results

**SI Table 3. Trustworthiness ratings by condition and time**

| Time | Condition | Mean | SD subj |
| --- | --- | --- | --- |
| Pre | Arab | 4.16 | 0.96 |
|  | White | 4.59 | 0.88 |
|  |  |  |  |
|  | Arab Fair | 4.34 | 1.13 |
|  | White Fair | 4.65 | 1.01 |
|  | Arab Unfair | 3.98 | 1.01 |
|  | White Unfair | 4.53 | 1.06 |
|  |  |  |  |
| Post | Fair | 4.70 | 0.94 |
|  | Unfair | 3.49 | 0.90 |
|  |  |  |  |
|  | Arab Fair | 4.72 | 1.34 |
|  | White Fair | 4.69 | 1.18 |
|  | Arab Unfair | 3.66 | 1.11 |
|  | White Unfair | 3.32 | 1.32 |

Mean first aggregated by subject; Mean and SD were then calculated across subjects.

**SI Table 4. Trustworthiness Ratings Correlations for Dependent t-tests**

| **Time** | Contrast | Correlation | **Time** | Contrast | Correlation |
| --- | --- | --- | --- | --- | --- |
| **Pre** | Arab v. White | 0.23 | **Change**  **(post-pre)** | Fair Arab | 0.50 |
|  | Fair Arab v. White | 0.30 |  | Unfair Arab | 0.12 |
|  | Unfair Arab v. White | 0.11 |  | Fair White | 0.29 |
| **Post** | Fair v. Unfair | -0.13 |  | Unfair White | 0.31 |
|  | Fair White v. Arab | 0.10 |  |  |  |
|  | Unfair White v. Arab | 0.11 |  |  |  |

The ratings were first aggregated on a subject level, then a correlation was calculated for n=73.

## Color and Learning Results

To test the possibility that the color assigned to a particular partner ethnicity-fairness combination during learning in the multi-round trust game, we added color as a covariate to the mixed effects model as described in Behavioral Analyses - Trustworthiness Ratings Pre vs. Post TG section in the main text. There was no significant effect of color, and the interaction of partner fairness and ethnicity remained significant, *β* = -0.55, *t* = -2.26, p = 0.027.

# Sensitivity Analyses for Trustworthiness Ratings Linear Mixed Model

The sensitivity analysis for the linear mixed model was done using simulations in the *mixedpower* package in R (Kumle, Vo, & Draschkow, 2021). The algorithm estimates the power of each individual effect in a linear mixed model. To focus on the interaction effect and estimate the expected effect size given power of 80% and a fixed sample size of 73, a wide range of effect sizes was selected (0.2 to 0.8 in steps of 0.1). For each of these effect sizes, 1000 simulations were conducted to estimate the power associated with each effect size. Then, a narrower range of effect sizes clustered around 80% power were selected and tested again with 1000 simulations.

To assess the sensitivity of the interaction effect of partner fairness and partner ethnicity on change in trustworthiness ratings, we did the following: holding all other effect sizes in the model constant, we estimated the power 1000 times for interaction effect sizes of -0.2 to -0.8 in steps of 0.1. An effect of -0.6 yielded 75.6% power, and an effect of -0.7 yielded 85.1% power. We followed up with a similar analysis of power associated with effect sizes of -0.6 to -0.7 in steps of 0.01. To reach power of 80% with a sample of 73 for this linear mixed model, an interaction effect of partner fairness and ethnicity of -0.65 or greater is needed. Results are in the sensitivity results subfolder of the results repository.

# Transfer Task

## **SI Table 5. Two-ethnicity comparison**

N obs: 10508, groups: image, 159; subject ID, 73

The model with random slopes for image ethnicity and ethnicity percent by subject ID failed to converge. As such, this model contains random intercepts, but not random slopes, for subject and image.

| **Fixed Effects** | **Estimate** | **Std Error** | **t-value** | **p-value** |
| --- | --- | --- | --- | --- |
| Intercept | 0.51 | 0.47 | 1.09 | 0.28 |
| Image ethnicity | 0.03 | 0.10 | 0.26 | 0.80 |
| Ethnicity percent | -0.62 | 0.49 | -1.28 | 0.20 |

**Correlation of Fixed Effects**

|  | Intercept | Ethnicity-Middle Eastern |
| --- | --- | --- |
| Ethnicity-Middle Eastern | -0.83 |  |
| Ethnicity percent | -0.99 | 0.80 |

| **Random Effects** | **Type** | **Variance** | **Std Dev.** |  |
| --- | --- | --- | --- | --- |
| Image | Intercept | 0.14 | 0.37 |  |
| Subject ID | Intercept | 0.19 | 0.44 |  |

## **SI Table 6. Three-ethnicity comparison**

N obs: 10508, groups: image, 159, subject ID, 73

| **Fixed Effects** | **Estimate** | **Std. Error** | **t-value** | **p-value** |
| --- | --- | --- | --- | --- |
| Intercept | 0.42 | 0.46 | 0.93 | 0.36 |
| Turkish | 0.09 | 0.11 | 0.81 | 0.42 |
| Moroccan | -0.23 | 0.15 | -1.54 | 0.12 |
| Ethnicity Percent | -0.53 | 0.48 | -1.10 | 0.27 |

**Correlation of Fixed Effects**

|  | Intercept | Turkish | Moroccan |
| --- | --- | --- | --- |
| Turkish | -0.74 |  |  |
| Moroccan | -0.53 | 0.63 |  |
| Ethnicity Percent | -0.99 | 0.69 | 0.48 |

| **Random Effects** | **Type** | **Variance** | **Std. Dev.** |
| --- | --- | --- | --- |
| Image | Intercept | 0.13 | 0.37 |
| Subject ID | Intercept | 0.34 | 0.59 |
|  | Turkish | 0.16 | 0.40 |
|  | Moroccan | 0.29 | 0.54 |
|  | Ethnicity Percent | 0.37 | 0.62 |

**Correlation of Random Effects**

|  | Subject Intercept | Slope: Turkish | Slope: Moroccan |
| --- | --- | --- | --- |
| Slope: Turkish | 0.01 |  |  |
| Slope: Moroccan | 0.03 | 0.82 |  |
| Slope: ethnicity percent | -0.66 | -0.32 | -0.35 |

# Transfer Task Follow-up Study

## **SI Table 7. Three-ethnicity comparison**

N obs: 12686, groups: image 158, subject ID 83

| **Random Effects** | **Type** | **Variance** | **Std. Dev** |
| --- | --- | --- | --- |
| Image | Intercept | 0.12 | 0.35 |
| Subject ID | Intercept | 0.44 | 0.67 |
|  | Turkish | 0.24 | 0.48 |
|  | Moroccan | 0.24 | 0.49 |
|  | Ethnicity Percent | 0.39 | 0.63 |
| **Fixed Effects** | **Estimate** | **Standard Error** | **t-value (p-value)** |
| Intercept | 0.51 | 0.44 | 1.16 (.25) |
| Turkish | -0.06 | 0.11 | -0.52 (.61) |
| Moroccan | -0.28 | 0.14 | -2.06 (.04) |
| Ethnicity Percent | -0.53 | 0.46 | -1.17 (.25) |

**Correlation of Random Effects**

|  | Subject Intercept | Slope: Turkish | Slope: Moroccan |
| --- | --- | --- | --- |
| Slope: Turkish | -0.27 |  |  |
| Slope: Moroccan | 0.10 | 0.77 |  |
| Slope: ethnicity percent | -0.62 | -0.07 | -0.47 |

**Correlation of Fixed Effects**

|  | Intercept | Turkish | Moroccan |
| --- | --- | --- | --- |
| Turkish | -0.73 |  |  |
| Moroccan | -0.53 | 0.62 |  |
| **Ethnicity Percent** | **-0.99** | **0.67** | **0.48** |

# Reinforcement Learning Models

## Model Selection

### Block analysis

All models were fit for each block to examine potential block differences. For blocks 2 and 3, the values for the participant’s expected probability of a partner reciprocating (*ep)* for each partner at the end of the former block were used to initialize the *ep* values in the present block. This was done to account for the learning that already occurred.

The simple learn model had the lowest mean BIC across participants in each individual block (SI Table 8). It also had the lowest BIC for the greatest number of participants in all blocks (SI Figure 1). Of note is that as the experiment progressed, more participants’ behavior fit the NL optimal model, suggesting they stopped learning and began investing optimally. However, the simple learn model still fits most participants best, and provides the best fit across the full experiment.

**SI Table 8. Mean Model BIC for individual blocks.**

| **Block** | NL Bias | NL Optimal | Simple Learn | Reputation | LG | LGK | L2G2 |
| --- | --- | --- | --- | --- | --- | --- | --- |
| **1** | 67.5 (5.8) | 59.4 (14.8) | 58.1 (13.0) | 63.5 (13.2) | 62.7 (14.8) | 67.2 (13.7) | 68.5 (14.9) |
| **2** | 66.6 (8.6) | 49.3 (22.1) | 46.2 (20.5) | 52.2 (21.7) | 52.8 (21.7) | 56.0 (21.7) | 58.4 (21.4) |
| **3** | 67.0 (9.6) | 42.8 (23.9) | 39.7 (22.4) | 48.4 (22.7) | 48.3 (22.4) | 52.8 (22.7) | 54.6 (22.0) |

M (SD)


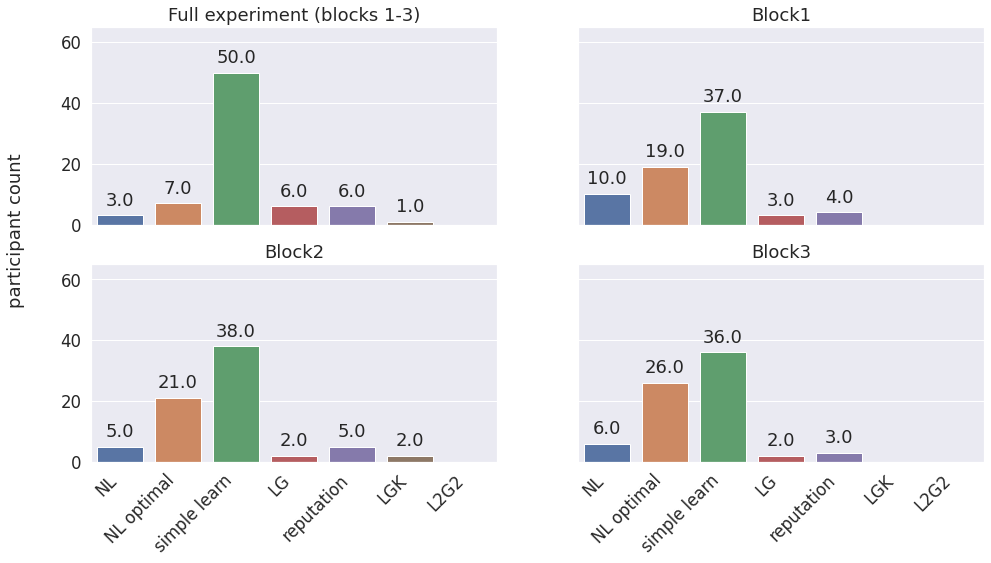


**SI Figure 1**. Number of participants for whom each model had the lowest BIC by block.

## Model Validation

While the BIC-based evaluations provide some information about the model’s explanatory power for the participants’ behavior, it is necessary to check how well those models match participants’ behavior through model validation procedures (Zhang et al., 2020).

Consequently, we simulated 100 data sets for each participant for the simple learn, LG, and reputation models. The optimal parameters estimated for each individual participant for the composite block (whole experiment) were used to simulate the data. Participants’ simulated choices were done probabilistically: within each model, there is an internal variable of the probability of a participant investing on a given trial. The participant’s choice of “invest” or “keep” was determined in the simulation using this value, e.g. if the probability of a participant investing for a given trial was 0.7, then the choice “invest” was selected with a probability of 0.7.

When averaging across all participants, the model simulations produce similar patterns (SI Figure 2). However, the reputation model underestimates the investment behavior in later trials. The LG and simple learn models are similar, with learning being steeper in earlier trials for the simple learn model compared to the LG model. Although all models overestimate the likelihood of investment on the first trial (around 0.6 compared to the actual of 0.5), the LG model overestimates this the most. Considering this, we still conclude that the simple learn model provides a good qualitative fit of participants’ investment behavior, with the other two being solid alternatives.


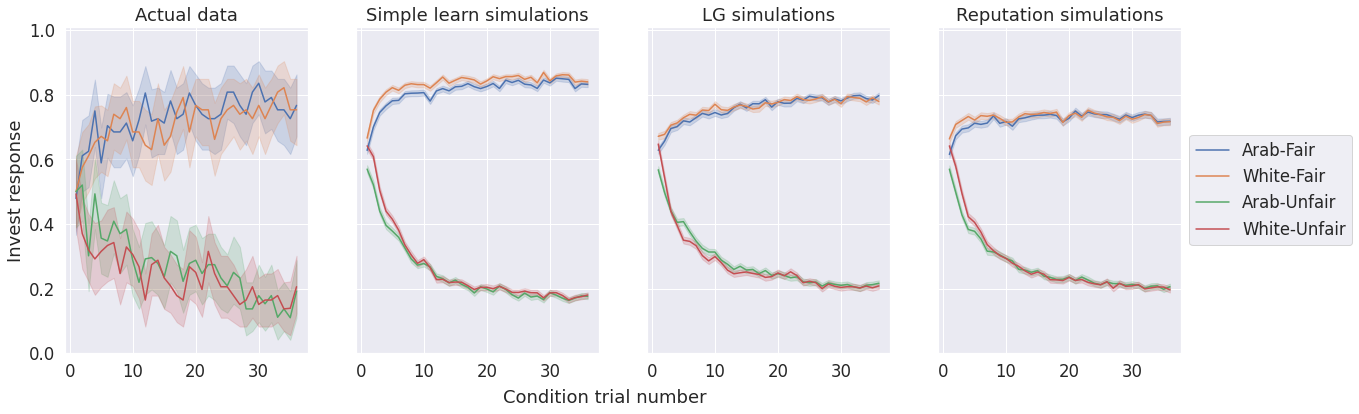


**SI Figure 2.** A comparison of the participants’ actual investment responses with each partner type in the experiment with invest responses estimated by the 100 stimulations for each participant, for the simple learn, reputation and LG models. For participants’ actual data, the proportion of invest responses made by all participants at a given condition trial were calculated. For simulations, this was calculated the same way, except not only across all participants but all simulations as well. The shaded areas represent 95% confidence intervals.

To evaluate the models further, we selected 3 participants who each exhibited a different type of learning behavior in the multi-round TG and examined how well the simple learn, LG, and reputation models predicted their behavior (SI Figure 3).

Depending on the participant, a different model may fit their behavior best. For example, with the decent learner (SI Figure 3A), the simple learn model overestimates the learning, whereas the LG and reputation models capture better how the participant invests on later trials. With the good learner (SI Figure 3B), all models capture the general investment pattern, but the simple learn model captures the low investments with unfair partners in the earlier blocks, in addition to the investment end points. With the bad learner, none of the models accurately capture the investments with the Fair-Arab partner, although all do a good job for the remaining conditions. Taken together, this shows that qualitatively, on a case-by-case basis, the models do a similar job of capturing the behavior, with some advantages and disadvantages. This indicates that the model that quantitatively has the best fit is a reliable metric and considering the qualitative fit at the group level.

This coupled with the results that the simple learn model had the lowest BIC for the majority of participants, confirms it as the winning model.

### Comparison of Different Types of Learners

A.


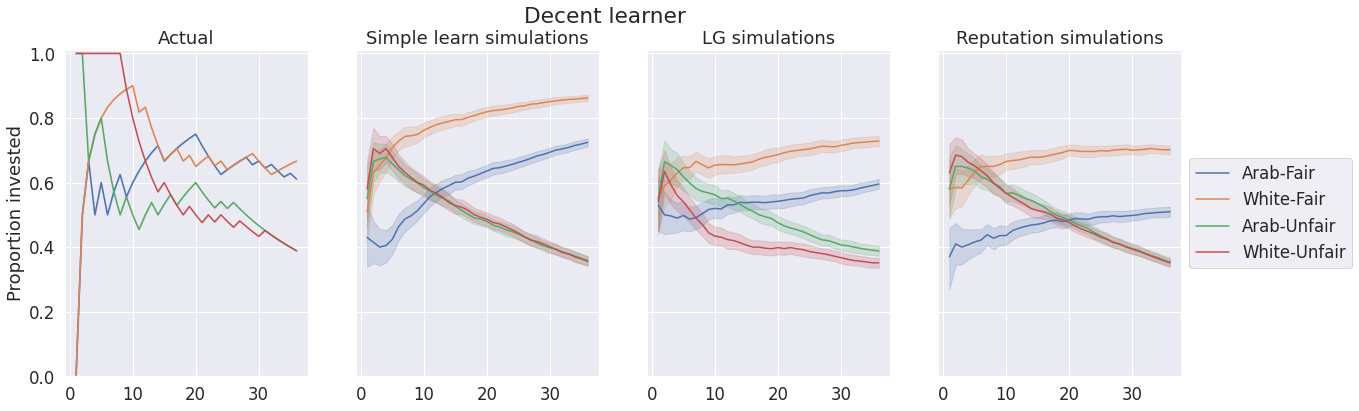


B.
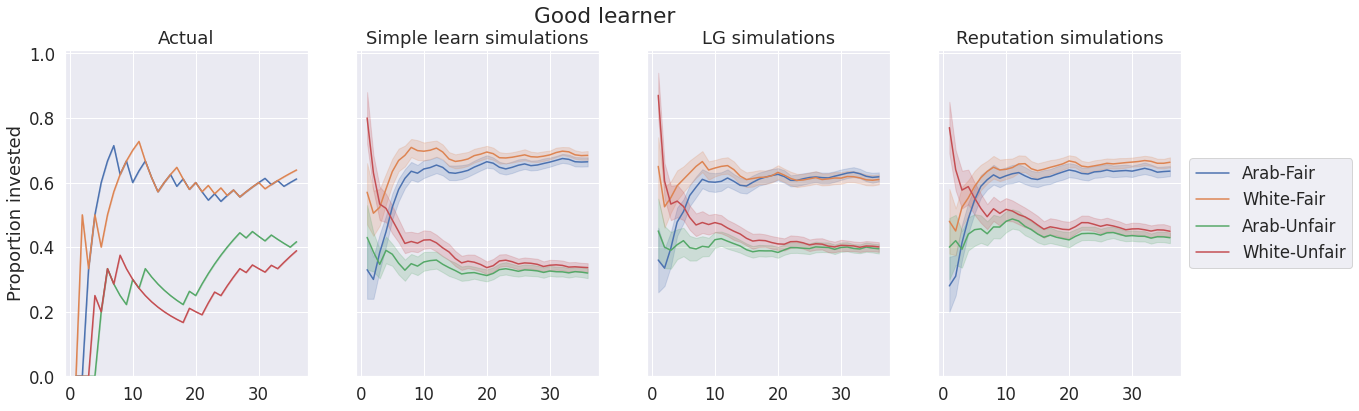


C.
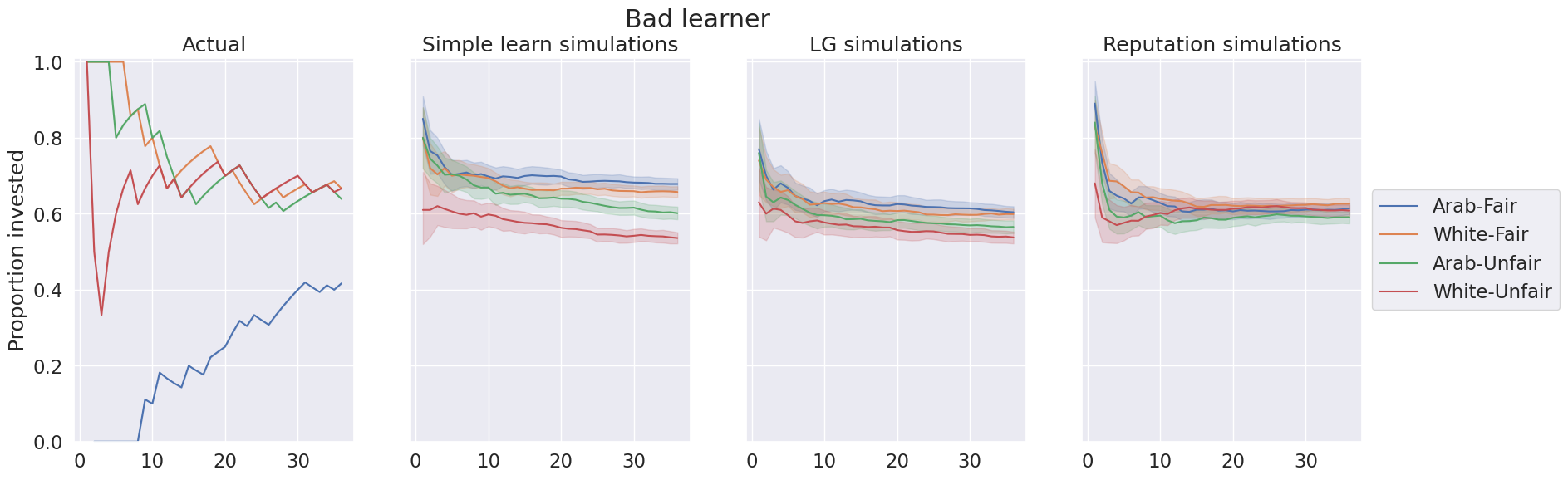


**SI Figure 3**. A comparison of participants’ actual proportion of invest trials with that predicted by the simple learn, LG, and Reputation models for three different participants, each of whom exhibited a different learning pattern in the multi-round TG. The proportion invested was calculated as the ratio of invest decisions made at each trial within each condition. (**A)** An example of a decent learner – one who learned to invest more with fair partners than with unfair partners by the end of the experiment. (**B)** An example of a good learner – one who learned to invest more with fair partners than with unfair partners in block 1 and maintained that until the end of the experiment. (**C)** An example of a bad learner. This participant did not invest more with fair partners compared to unfair partners; in fact, they invested less with the fair Arab partner compared to the rest. The shaded areas represent 95% confidence intervals.

## Simple learn ethnicity and fairness models

As part of the follow-up analysis to evaluate if participants learned from their different partners differently, the simple learn ethnicity and simple learn fairness models were conducted. The mean BIC (SI Table 9; SI Figure 4A) and frequency of lowest BIC across participants was compared (SI Figure 4B).

**SI Table 9. Simple Learn Models, Estimated Parameters and BIC values**

|  | Simple Learn | Ethnicity | Fairness |
| --- | --- | --- | --- |
| BIC | 134.1 (50.3) | 156.4 (52.8) | 154.10 (52.4) |
| β | 0.62 (0.45) | 2.47 (9.45) | 1.20 (1.79) |
| α | 0.16 (0.10) | A: 0.23 (0.23)  W: 0.18 (0.17) | F: 0.10 (0.15)  U: 0.22 (0.18) |

Mean (SD)

1. **B.**


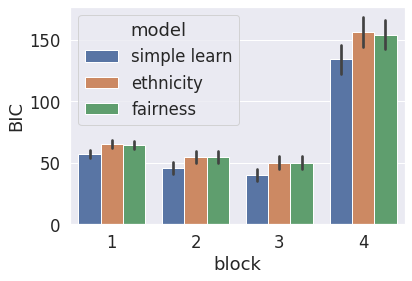

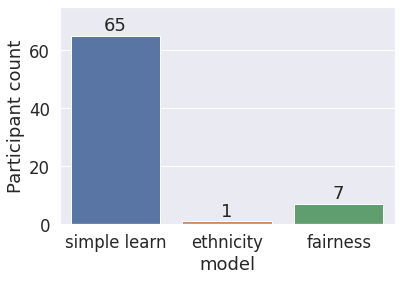


**SI Figure 4.** A. Mean BIC by block. Black bars are 95% confidence intervals. Block 4 refers to the composite experiment. B. Number of participants for whom each model had the lowest BIC.

The results demonstrate that the simple learn model had the lowest mean BIC (SI Figure 4A), as well as having the lowest BIC for the greatest number of participants (SI Figure 4B) compared to the other two models, confirming its robustness as the best fit model.

There was one participant for whom the simple learn ethnicity model was the best fit. With only 1 data point, it is not possible to conduct a t-test on the learning rates for Arab and White partners. Similarly, for participants for whom the simple learn fairness model was the best fit (*n* = 7), a dependent t-test revealed that there was a no statistically significant difference between the learning rate for fair partners (*M* = 0.12, *SD* = 0.14) and unfair partners (*M* = 0.09, *SD* = 0.10), *t*(6) = 0.37, *p* = 0.73, *Hedges’ g* = 0.23 95% CI [-1.21, 1.77].

Although the simple learn model which reflected most participants’ learning process, when learning rates for different partner types were allowed, for example with the simple learn fairness model, those learning rates did not differ significantly. This provides further evidence that the participants’ behavior was sufficiently captured with one learning rate.

# References

Gawronski, B (2002). What does the Implicit Association Test Measure? A Test of the

Convergent and Discriminant Validity of Prejudice-Related IATs. *Experimental Psychology,* *49*(3), 171-180 [https://doi.org/10.1026/1618-3169.49.3.171](https://psycnet.apa.org/doi/10.1026/1618-3169.49.3.171)*.*

Hansen, B.B. and Klopfer, S.O. (2006). Optimal full matching and related designs via network

flows, Journal of Computational and Graphical Statistics, *15*, 609-627. <https://doi.org/10.1198/106186006X137047>

Kumle, L., Vo, M.L., & Draschkow, D. (2021). Estimating power in (generalized) linear mixed

models: an open introduction and tutorial in R. *Behavioral Research*. <https://doi.org/10.3758/s13428-021-01546-0>

Langner, O., Dotsch, R., Bijlstra, G., Wigboldus, D. H. J., Hawk, S.T., and van Knippenberg, A.

(2010). Presentation and validation of the Radboud Faces Database. *Cognition and Emotion*, *24*(8), 1377-1388. <https://doi.org/10.1080/02699930903485076>

Ma, D.S, Correll, J., and Wittenbrink, B. (2015). The Chicago face database: A free stimulus set

of faces and norming data. *Behavioral Research*, *47*, 1122-1135.

<https://doi.org/10.3758/s13428-014-0532-5>

Nosek, B.A., Smyth, F.L. Hansen, J.J., Devos, T., Lindner, N.M., Ranganath, K.A., Tucker

Smith, C., Olson, K.R., Chugh, D., Greenwald, A.G., & Banaji, M.R. (2007). Pervasiveness and correlates of implicit attitudes and stereotypes. *European Review of Social Psychology,* *18*(1), 1-53. <https://doi.org/10.1080/10463280701489053>

Saribay, S.A., Biten, A.F, Meral, E.O., Aldan, P., Trebicky, V., & Kleisner, K. (2018). The

Bogazici face database: Standardized photographs of Turkish faces with supporting materials. *PLoS ONE*, *13*(2), e0192018. https://doi.org/10.1371/journal.pone.0192018

Zhang, L., Lengersdorff, L., Mikus, N., Glaescher, J., & Lamm, C. (2020). Using reinforcement

learning models in social neuroscience: frameworks, pitfalls and suggestions of best practices. *Social Cognitive Affective Neuroscience*, *15*(6), 695-707.

<https://doi.org/10.1093/scan/nsaa089>
